# Supplementary material for: Economic Analysis of Border Control Policies during COVID-19 Pandemic: A Modelling Study to Inform Cross-Border Travel Policy between Singapore and Thailand
Source: Int J Environ Res Public Health. 2023 Feb 23;20(5):4011. doi: 10.3390/ijerph20054011 (PMC10001629; doi:10.3390/ijerph20054011)
Supplement: Supplementary file 1 [file ijerph-20-04011-s001.zip › File S1.pdf]

Supporting Information S1. Input value and data source of parameters

Table of Contents

Supporting Information S1. Input value and data source of parameters........2

Table S1 Parameters on economics and tourism........2

Table S2 Parameters on COVID-19 measures........3

Table S3 Risk profile of COVID-19 patients and vaccine efficacy........4

Table S4 Parameters on cost related to COVID-19 cases........5

Table S5 Parameters on health outcome........6

Table S6 Parameters on COVID-19 transmission........7

References........8

Table S1 Parameters on economics and tourism

Parameter description	Value	Data source
Per capita expenditure of tourist from Thailand (TH) in Singapore (SG)	US$811·53	Singapore Tourism Board (STB)
Per capita expenditure of business traveler from TH in SG	US$1623·06	Assumed as twice of tourist from TH in SG
Per capita expenditure of tourist from SG in TH	US$1028·72	Tourism Authority of Thailand (TAT)
Per capita expenditure of business traveler from SG in TH	US$2057·44	Assumed as twice of tourist from SG in TH
Aviation spending per traveler for a round trip between SG and TH	US$251·60	Singapore Airlines, Scoot and AirAsia
Length of stay in TH for travelers from SG	5·54 days	TAT
Daily consumption expenditure of SG residents if not travelling	US$37·36	Department of Statistics (DOS), Singapore
Length of stay in SG for travelers from TH	3·46 days	STB
Daily consumption expenditure of TH residents if not travelling	US$10·22	National Statistical Office (NSO), Thailand
Monthly income of population in SG	US$3174·00	Ministry of Manpower (MOM), Singapore
Monthly income of population in TH	US$500·00	Minimum wage rate in Thailand, suggested by Health Intervention and Technology Assessment Program (HITAP), Thailand
Cost effectiveness threshold of SG	US$59797·75	World Bank, assumed to equal 1 GDP per capita
Cost effectiveness threshold of TH	US$7189·40	
Cost effectiveness threshold of SG (DSA)	US$17393·25	World Bank, assumed to equal 3 GDP per capita
Cost effectiveness threshold of TH (DSA)	US$21568·20	
Tourism multiplier in SG (DSA)	2.35	Estimated based on input-output table[1] and tourism receipt component[2] of Thai travelers in Singapore
Tourism multiplier in TH (DSA)	2.09	Sindechara[3]

Table S2 Parameters on COVID-19 measures

Parameter description	Value	Data source
Daily price of quarantine at SG	US$107·14	Immigration & Checkpoints Authority (ICA) and Ministry of Health (MOH), Singapore
Daily price of quarantine at TH	US$79·88	Spot check on hotels in TH
Percentage productivity loss if quarantined	30%	Ballegooijen et al.[4]
Percentage productivity loss if quarantined (DSA)	0%	Assume no productivity loss due to quarantine/ isolation
Antigen sensitivity by log-viral load, logit constant coefficient	-3·748627	Peto et al.[5]
Antigen sensitivity by log-viral load, logit linear coefficient	1·080585	
PCR sensitivity by log-viral load, logit constant coefficient	-2·67331	Miller et al.[6]
PCR sensitivity by log-viral load, logit linear coefficient	0·929224	
Cost of ART test in SG	US$7·5	Estimated based on advice by MOH, Singapore
Cost of PCR pre-test and confirmation test in SG	US$56·25	
Cost of PCR test upon arrival at SG	US$60	
Cost of PCR test during and exit quarantine in SG	US$46·88	
Cost of ART test in TH	US$10	Estimated based on advice by HITAP, Thailand
Cost of conducting PCR test in TH	US$34·75	
Administrative cost for test-trace-isolation (TTI) cost associated with one more COVID-19 case in SG	US$3071·10	Derived based on labor cost of TTI staff (source: MOH, Singapore; Indeed) and testing cost.
Administrative cost for TTI associated with one more COVID-19 case in TH	US$0·00	No more contact tracing at the time of analysis.

Table S3 Risk profile of COVID-19 patients and vaccine efficacy

Parameter description		Value	Data source
Unvaccinated local/ secondary cases in SG:	Probability of asymptomatic	36·94%	Derived based on an age-specific risk profile (source: Oran et al.[7], Wei et al.[8], O'Driscoll et al.[9], Sheikh et al.[10],) and different age structures of:<br>- SG general population (source: DOS, Singapore)<br>- TH general population (source: PopulationPyramid.Net)<br>- travelers from SG to TH (source: TAT, Thailand)<br>- travelers from TH to SG (source: STB, Singapore)
	Probability of mild/ moderate	50·55%	
	Probability of severe	9·56%	
	Probability of critical	2·06%	
	Probability of death	0·89%	
Unvaccinated local/ secondary cases in TH:	Probability of asymptomatic	37·93%	
	Probability of mild/ moderate	50·65%	
	Probability of severe	8·75%	
	Probability of critical	1·86%	
	Probability of death	0·81%	
Unvaccinated cases traveling from SG to TH:	Probability of asymptomatic	36·94%	
	Probability of mild/ moderate	53·67%	
	Probability of severe	7·35%	
	Probability of critical	1·48%	
	Probability of death	0·56%	
Unvaccinated cases traveling from TH to SG:	Probability of asymptomatic	37·93%	
	Probability of mild/ moderate	54·94%	
	Probability of severe	5·68%	
	Probability of critical	1·10%	
	Probability of death	0·34%	
Vaccine in SG	Coverage in general population	84%	MOH, Singapore, as in Oct 2021
	Coverage in general population (DSA)	92%	MOH, Singapore, as in Jan 2023
	Percentage reduction against infection	69%	National Center of Infectious Disease (NCID), Singapore
	Percentage reduction against symptomatic infection	80%	Public Health England[11]
	Percentage reduction against severe/critical cases	95%	Stowe et al.[12]
Vaccine in TH	Coverage in general population	35%	Ministry of Public Health (MoPH), Thailand, as in Oct 2021
	Coverage in general population (DSA)	76%	Ministry of Public Health (MoPH), Thailand, as in Jan 2023
	Percentage reduction against infection	50%	AstraZeneca[13]
	Percentage reduction against symptomatic infection	79%	
	Percentage reduction against severe/critical cases	95%	

Table S4 Parameters on cost related to COVID-19 cases

Parameter description		Value	Data source
Cost of treating an unvaccinated case in SG	asymptomatic	US$78·75	Derived based on data shared by MOH and NCID, Singapore, and HITAP, Thailand, taking into account cost of hospitalization, tests, and transport.
	mild/moderate	US$91·25	
	severe	US$6937·50	
	critical	US$22650·00	
Cost of treating a vaccinated case in SG	asymptomatic	US$71·25	
	mild/moderate	US$81·25	
	severe	US$4912·50	
	critical	US$16500·00	
Cost of treating an unvaccinated case in TH	asymptomatic	US$34·75	
	mild/moderate	US$34·75	
	severe	US$3469·30	
	critical	US$16451·57	
Cost of treating a vaccinated case in TH	asymptomatic	US$34·75	
	mild/moderate	US$34·75	
	severe	US$3469·30	
	critical	US$16451·57	
Cost of treating a unvaccinated/ vaccinated case in SG/ TH (DSA)		-	Double all the base case values above
Percentage productivity loss if hospitalized		100%	Assumed
Percentage productivity loss of asymptomatic cases		30%	Assumed to be the same as productivity loss due to quarantine.
Percentage productivity loss of asymptomatic cases (DSA)		0%	
Percentage productivity loss of mild/moderate cases		100%	Assume symptomatic cases need to rest without working

Table S5 Parameters on health outcomes

Parameter description		Value	Data source
Quality of life (QoL) of general population (SG and TH)		0·95	Abdin et al.[14]
Percentage reduction in QoL due to isolation		2·92%	Wong et al.[15]
Quality-adjusted life years (QALY) loss of an asymptomatic case:	Unvaccinated, isolated in SG	0·000684	Estimated based on length of isolation.
	Vaccinated, isolated in SG	0·000456	
	Unvaccinated, isolated in TH	0·00213	
	Vaccinated, isolated in TH	0·00213	
QALY loss of an unvaccinated case:	Mild/moderate	0·43	Basu et al.[16]
	Severe	0·50	
	Critical	0·60	
Percentage QALY loss saved for symptomatic cases if vaccinated		10%	Assumed
Percentage QALY loss saved for symptomatic cases if vaccinated (DSA)		0%	Assume vaccinated and unvaccinated cases have same QALY loss upon showing symptoms
QALY loss of all the close contacts of one case due to quarantine in SG		0·00127	Estimated based on length of isolation.
QALY loss of all the close contacts of one case due to quarantine in TH		0	
QALY loss of COVID death in SG		8·62	Estimated based on average age of COVID-19 mortality and life expectancy. Discounted by 3%.
QALY loss of COVID death in TH		3·76	

Table S6 Parameters on COVID-19 transmission

Parameter description	Value	Data source
Reproduction rate (R0) without effect of vaccines	7	High estimate for Delta
R0 without effect of vaccines (DSA)	10	High estimate for Omicron
Daily infection rate in SG among general population	0·00059	WHO<br>Worldometers<br>MOH, Singapore
Daily infection rate in TH among general population	0·00014	
Daily infection rate in SG among general population (DSA)	0.000885	Increase by 50% from base case values
Daily infection rate in TH among general population (DSA)	0.00021	
Prevalence in SG	0·0059	Derived by assuming daily infection rate equals 1/10 of prevalence, i.e. the duration of disease is 10 days[17]
Prevalence in TH	0·0014	
Prevalence in SG (DSA)	0.00885	Increase by 50% from base case values
Prevalence in TH (DSA)	0.0021	
Mean slope of increasing log-viral load	2·806329	Jones et al.[18]
Standard deviation (SD) of slope of increasing log-viral load	0·39	
Mean slope of decreasing log-viral load	-0·39152	
SD of slope of decreasing log-viral load	0·02	
Mean peak log-viral load	10·0635	
SD of peak log-viral load	0·7	
Mean days to peak log-viral load	4·3	
SD of days to peak log-viral load	0·92	
Mean days from peak log-viral load to symptoms	4·3	
SD of days from peak log-viral load to symptoms	0·488	

References

1. Department of Statistics Singapore. National Accounts Input-Output Tables. 2017. Available online: https://www.singstat.gov.sg/find-data/search-by-theme/economy/national-accounts/latest-data (accessed on 18 February 2023).

2. Singapore Tourism Analytics Network. Tourism Receipts Statistics. Available online: https://stan.stb.gov.sg/content/stan/en/home.html (accessed on 18 February 2023).

3. Sindechara, T. Economic Import Evaluation from Tourism in Designated Area for Sustainable Tourism. 2017. Available online: https://data.go.th/dataset/ac3db6c0-2f8c-444d-b639-1008569ff62e/resource/3699e612-115e-4014-a941-afa80454bfbc/download/multiplier-effects.pdf (accessed on 18 February 2023).

4. Ballegooijen, H.; Goossens, L.; Bruin, R.H.; Michels, R.; Krol, M. Concerns, quality of life, access to care and productivity of the general population during the first 8 weeks of the coronavirus lockdown in Belgium and the Netherlands. *BMC Health Serv. Res.* 2021, 21, 1–8.

5. Peto, T.; UK COVID-19 Lateral Flow Oversight Team. COVID-19: Rapid antigen detection for SARS-CoV-2 by lateral flow assay: A national systematic evaluation of sensitivity and specificity for mass-testing. *EClinicalMedicine* 2021, 36, 100924.

6. Miller, T.E.; Miller, T.E.; Garcia Beltran, W.F.; Bard, A.Z.; Gogakos, T.; Anahtar, M.N.; Astudillo, M.G.; Yang, D.; Thierauf, J.; Fisch, A.S.; Mahowald, G.K.; et al. Clinical sensitivity and interpretation of PCR and serological COVID-19 diagnostics for patients presenting to the hospital. *FASEB J. 2020,* 34, 13877–13884.

7. Oran, D.P.; Topol, E.J. Prevalence of asymptomatic SARS-CoV-2 infection: a narrative review. *Ann. Intern. Med*. 2020, 173, 362–367.

8. EW, W.; Wycliffe, W.E.; Heng, T.C.; Chan, M.; Tong, T.T.; Pada, S.K.; Archuleta, S.; Tat, O.S.; Jiashen, L.; Choon, R.F.K.; Cheng, T.K.; et al. Age and chest radiography as possible parameters for rapid triage in COVID-19 outbreak surge. 2020. https://doi.org/10.21203/rs.3.rs-88104/v1

9. O'Driscoll, M.; Santos, G.R.D.; Wang, L.; Cummings, D.A.T.; Azman, A.S.; Paireau, J.; Fontanet, A.; Cauchemez, S.; Salje, H. Age-specific mortality and immunity patterns of SARS-CoV-2. *Nature* 2021, 590, 140–145.

10. Sheikh, A.; McMenamin, J.; Taylor, B.; Robertson, C. SARS-CoV-2 Delta VOC in Scotland: demographics, risk of hospital admission, and vaccine effectiveness. *Lancet* 2021, 397, 2461-2462.

11. *SARS-Cov-2 Variants of Concern and Variants under Investigation in England*; Public Health England: London, UK, 2021.

12. Lopez Bernal J, Andrews N, Gower C, Gallagher E, Simmons R, Thelwall S, Stowe J, Tessier E, Groves N, Dabrera G, *et al.* Effectiveness of COVID-19 vaccines against hospital admission with the Delta (B.1.617.2) variant. *N. Engl. J. Med.* 2021, 388, 672.

13. AstraZeneca. AZD1222 US Phase III Trial Met Primary Efficacy Endpoint in Preventing COVID-19 at Interim Analysis. 2021. Available online: https://www.astrazeneca.com/media-centre/press-releases/2021/astrazeneca-us-vaccine-trial-met-primary-endpoint.html (accessed on 15 July 2021).

14. Abdin, E.; Subramaniam, M.; Vaingankar, J.A.; Luo, N.; Chong, S.A. Population norms for the EQ-5D index scores using Singapore preference weights. *Qual. Life Res.*2015, 24, 1545–1553.

15. Wong, E.L.; Ho, K.F.; Wong, S.Y.; Cheung, A.W.; Yau, P.S.; Dong, D.; Yeoh, E.K. Views on workplace policies and its impact on health-related quality of life during coronavirus disease (COVID-19) pandemic: cross-sectional survey of employees. *Int. J. Heal. policy Manag.* 2022, 11, 344-353.

16. Basu, A.; Gandhay, V.J. Quality-Adjusted Life-Year Losses Averted With Every COVID-19 Infection Prevented in the United States. *Value Heal.* 2021, 24, 632–640.

17. Herrero, L. How Contagious Is Delta? How Long Are You Infectious? Is It More Deadly? A Quick Guide to the Latest Science. The Conversation. Available online: https://theconversation.com/how-contagious-is-delta-how-long-are-you-infectious-is-it-more-deadly-a-quick-guide-to-the-latest-science-165538 (accessed on 15 October 2021).

18. Jones, T.C.; Biele, G.; Mühlemann, B.; Veith, T.; Schneider, J.; Beheim-Schwarzbach, J.; Bleicker, T.; Tesch, J.; Schmidt, M.L.; Sander, L.E.; *et al.* Estimating infectiousness throughout SARS-CoV-2 infection course. *Science* 2021, 373, eabi5273.
