# Supplementary material for: Economic Analysis of Border Control Policies during COVID-19 Pandemic: A Modelling Study to Inform Cross-Border Travel Policy between Singapore and Thailand
Source: Int J Environ Res Public Health. 2023 Feb 23;20(5):4011. doi: 10.3390/ijerph20054011 (PMC10001629; doi:10.3390/ijerph20054011)
Supplement: Supplementary file 1 [file ijerph-20-04011-s001.zip › File S2.pdf]

Supporting Information S2. Additional details about transmission model

The transmission model uses Monte Carlo simulation to estimate testing, quarantine and COVID-19 transmission outcomes for travelers from country A to country B. For each policy of testing and quarantine to be explored, a cohort of travelers initially infected in country A as well as a cohort of travelers only infected during their stay in country B are simulated, as described in manuscript section 2.4.

Vaccination rates and efficacies among travelers are accounted for in the simulation. To estimate the composition of vaccinated and unvaccinated among the travelers infected in country A, a Bayesian calculation is performed:

P(Vaccinated with V | Infected in country A)
= P(Infected in country A | Vaccinated with V) * P(Vaccinated with V) / P(Infected in country A)
= (Susceptibility of V)* P(Vaccinated with V) / ∑ (Susceptibility of V) * P(Vaccinated with V)

where susceptibility of V equals (1 – efficacy of V), and the sum ranges over the vaccines V (including no vaccine). To estimate the same composition among the travelers infected in country B (as well as their days of infection), a similar Bayesian calculation is first performed to determine the proportions of travelers not infected in country A that have given vaccines. Then, a Monte Carlo simulation is performed and the distribution of days of infection are derived for each vaccine (depending on its efficacy) by drawing from a geometric distribution, with daily probability of infection equal to the ratio of population infected per day in country B, multiplied by the ratio of the given vaccine's susceptibility to the average susceptibility in country B's general population.

Test sensitivities are computed depending on simulated viral loads, using estimated sensitivities by viral load. To simulate viral load profiles by day of infection, it is assumed that these profiles are described by a piecewise-linear function with one part linearly increasing from day 0 until a given day of peak load, and another linearly decreasing from day of peak load to the end of infection. The slopes, peak viral load, day of peak viral load and days from peak viral load to end of incubation period are assumed normally distributed with parameter values derived from a Jones et al.[1], but with adjustments for the higher peak viral load of Delta and correspondingly steeper slopes.

Reference

1 Jones, T.C.; Biele, G.; Mühlemann, B.; Veith, T.; Schneider, J.; Beheim-Schwarzbach, J.; Bleicker, T.; Tesch, J.; Schmidt, M.L.; Sander, L.E. Estimating infectiousness throughout SARS-CoV-2 infection course. *Science* 2021, 373, eabi5273.
