# Supplementary material for: Economic Analysis of Border Control Policies during COVID-19 Pandemic: A Modelling Study to Inform Cross-Border Travel Policy between Singapore and Thailand
Source: Int J Environ Res Public Health. 2023 Feb 23;20(5):4011. doi: 10.3390/ijerph20054011 (PMC10001629; doi:10.3390/ijerph20054011)
Supplement: Supplementary file 1 [file ijerph-20-04011-s001.zip › File S3.pdf]

Supporting Information S3. Additional details about testing and quarantine policies in Thailand and Singapore during the period Jan 2021 and Dec 2022

	**Thailand**	**Singapore**
2021		
January	Test: RT-PCR test within 72 hours before departure<br>Quarantine: 14 days<br>**Note:** this is only for people who have been vaccinated	Test: ART Test on arrival<br>Quarantine: N/A
February	Test: RT-PCR test within 72 hours before departure<br>Quarantine: 14 days	Test: PCR test on arrival<br>Quarantine: N/A
March	Test: RT-PCR test within 72 hours before departure<br>Quarantine: 14 days	Test: PCR test on arrival<br>Quarantine: N/A
April	Test: RT-PCR within 72 hours before departure<br>Quarantine: 7 days (Vaccinated travelers)	Test: PCR test within 72 hours before departure, on arrival, after 14 days and at the end of their 21 day quarantine period.<br>Quarantine: 21 days
May	Test: RT-PCR within 72 hours before departure<br>Quarantine: 7 days (Vaccinated travelers)	Test: PCR test within 72 hours before departure, upon arrival, and arrivals from most destinations will be issued with a Stay at Home Notice (SHN) for at least 21 days.<br>Quarantine: 21 days
June	Test: RT-PCR within 72 hours before departure<br>Quarantine: 7 days (Vaccinated travelers)	Test: PCR test within 72 hours before departure, upon arrival, during SHN<br>Quarantine: 7-14 days Stay-Home-Notice (SHN)<br>• travelers serving SHN in dedicated SHN facilities will be issued Antigen Rapid Test (ART) self-test kits and must undergo ART on the 3rd, 7th and 11th day of their arrival in Singapore.
July	Test: RT-PCR within 72 hours before departure and on arrival<br>Quarantine: 0 days if they entered the Phuket Sandbox Program	Test: PCR test within 72 hours before departure, upon arrival, during SHN<br>Quarantine: 7-14 days Stay-Home-Notice (SHN)<br>• Quarantine: travelers serving SHN in dedicated SHN facilities will be issued Antigen Rapid Test (ART) self-test kits and must undergo ART on the 3rd, 7th and 11th day of their arrival in Singapore.
August	Test: RT-PCR within 72 hours before departure and on arrival<br>Quarantine: 0 days if they entered the Phuket Sandbox Program	Test: PCR test within 72 hours before departure, upon arrival, during Quarantine<br>• At the end of the 14-day quarantine, a third PCR test will be conducted. Rapid tests are conducted on the third, seventh and eleventh days after entry.<br>Quarantine: 14 days

September	Test: RT-PCR within 72 hours before departure and on arrival<br>Quarantine: 0 days if they entered the Phuket Sandbox Program	Test: PCR test within 48 hours before departure, on arrival, up to two PCR tests during the stay.<br>Quarantine: 0 day (Enter country under Vaccinated Travel Lanes)
October	Test: RT-PCR within 72 hours before departure and on arrival<br>Quarantine: 0 days if they entered the Phuket Sandbox Program	Test: PCR test within 48 hours before departure, on arrival<br>Quarantine: 0 day<br>(Enter country under Vaccinated Travel Lanes)
November	Test: RT-PCR within 72 hours before departure and on arrival<br>Quarantine: 0 days if they entered Thailand under Sandbox Program in one of 17 provinces or Test and Go<br>Alternative quarantine: 7 days	Test: PCR/ART ART test within 48 hours before departure, day 7 of arrival<br>Quarantine: 0 day<br>(Enter country under Vaccinated Travel Lanes)
December	Test: RT-PCR within 72the hours before departure and on arrival<br>Quarantine: 0 days if they entered Thailand under the Phuket Sandbox Program Alternative quarantine: 7 days	Test: PCR/ART test within 48 hours before departure, day 7 of arrival<br>Quarantine: 0 day<br>(Enter country under Vaccinated Travel Lanes)
2022		
January	Test: RT-PCR within 72 hours before departure and on arrival<br>Quarantine: 0 days if they entered Thailand under Phuket Sandbox Program<br>Alternative quarantine: 10 days	Test: PCR/ART test within 48 hours before departure, on arrival<br>Quarantine: 0 day<br>(Enter country under Vaccinated Travel Lanes)
February	Test: RT-PCR within 72 hours before departure and on arrival<br>Quarantine: 0 days if they entered Thailand under Sandbthe ox Program or Test and Go<br>Alternative quarantine: 7 days	Test: PCR/ART test within 48 hours before departure<br>Quarantine: 0 day<br>(Enter country under Vaccinated Travel Lanes)
March	Test: RT-PCR within 72 hours before departure and on arrival<br>Quarantine: 0 days if they entered Thailand under the Sandbox Program or Test and Go<br>Alternative quarantine: 5 days	Test: PCR/ART test within 48 hours before departure<br>Quarantine: 0 days
April	Test: RT-PCR on arrival<br>Quarantine: 0 days if they entered Thailand under the Sandbox Program or Test and Go<br>Alternative quarantine: 5 days	Test: No test<br>Quarantine: 0 day
May	Test: No test before departure and on arrival<br>Quarantine: 0 day	Test: No test<br>Quarantine: 0 day
June	Test: No test before departure and on arrival<br>Quarantine: 0 day	Test: No test<br>Quarantine: 0 day
July	Test: RT-PCR or ATK within 72 hours before departure<br>Quarantine: 0 day	Test: No test<br>Quarantine: 0 day
August	Test: No test<br>Quarantine: 0 day	Test: No test<br>Quarantine: 0 day
September	Test: No test<br>Quarantine: 0 day	Test: No test<br>Quarantine: 0 day
October	Test: No test<br>Quarantine: 0 day	Test: No test<br>Quarantine: 0 day

November	Test: No test<br>Quarantine: 0 day	Test: No test<br>Quarantine: 0 day
December	Test: No test<br>Quarantine: 0 day	Test: No test<br>Quarantine: 0 day

Sources:

Thailand:

https://thailandpass.org/faq/

https://www.gov.uk/foreign-travel-advice/thailand/entry-requirements

https://maputo.thaiembassy.org/en/content/thailand-entry-measures-and-regulations?cate=5f4a21ad6dcd871a3466cf62

https://www.mfa.go.th/en/content/entry-measures-into-thailand-by-air-travel-0105202?cate=5f2916be298eeb02ac2eb389

https://oslo.thaiembassy.org/en/page/updated-measures-on-traveling-to-thailand?menu=6295fd96aa816330ea406663

https://www.caat.or.th/en/archives/56377

https://www.tatnews.org/2021/06/thailand-confirms-reopening-plan-from-1-july-2021/

https://www.mfa.go.th/en/content/thailand-pass-faqs

https://ankara.thaiembassy.org/en/publicservice/entry-to-thailand-during-covid-19?cate=618bb74713f3c853db3b4902

Singapore:

https://www.mfa.gov.sg/Overseas-Mission/Washington/Mission-Updates/2021/01/Update-on-COVID-19-on-15-Jan-2021

https://trutrip.co/singapore-travel-restrictions-update-feb-2021/

https://travelbans.org/en/asia/singapore#google_vignette

https://m.innovare-group.com/singapore/travel_and_quarantine_requirements/

https://www.visitsingapore.com/travel-guide-tips/travel-requirements/

https://mainlymiles.com/2021/07/27/singapore-aims-to-allow-quarantine-free-travel-from-september/

https://www.caas.gov.sg/who-we-are/newsroom/Detail/singapore-further-reopens-borders-in-cautious-step-by-step-manner

https://www.aviationpros.com/airports/news/21246133/spore-eases-border-measures-for-more-southeast-asian-countries-new-vtls-launched-with-finland-and-sweden

https://safetravel.ica.gov.sg/health/vtsg

https://www.ica.gov.sg/enter-depart/entry_requirements

https://www.singaporeair.com/en_UK/th/travel-info/covid-19/

https://www.moh.gov.sg/news-highlights/details/updates-on-border-measures-for-travellers-and-extending-vaccinated-travel-lanes

https://www.visitsingapore.com/th_th/travel-guide-tips/travel-requirements/

https://www.changiairport.com/en/airport-guide/Covid-19/travel-to-singapore.html

https://www.visitsingapore.com/travel-requirements/atp/

https://www.moh.gov.sg/news-highlights/details/further-easing-of-community-and-border-measures
